# Hyperthermic intraperitoneal chemotherapy enhances survival outcomes in primary ovarian cancer following cytoreductive surgery: a systematic review and meta-analysis

**DOI:** 10.3389/fonc.2025.1708318

**Published:** 2025-12-03

**Authors:** Qian Sun, Kangshuo Hu, Jingqi Chen, Yixia Chen, Zhongxuan Xie, Yuqing Guan, Yuqing Ying

**Affiliations:** 1 Department of Gynecology, Ningbo Yinzhou No. 2 Hospital, Ningbo, Zhejiang, China; 2 The Second School of Clinical Medical, Zhejiang Chinese Medical University, Hangzhou, Zhejiang, China; 3 The First School of Clinical Medical, Zhejiang Chinese Medical University, Hangzhou, Zhejiang, China; 4 The Fifth School of Clinical Medical, Zhejiang Chinese Medical University, Hangzhou, Zhejiang, China

**Keywords:** ovarian cancer, hyperthermic intraperitoneal chemotherapy, cytoreductive surgery, overall survival, progression-free survival, meta-analysis

## Abstract

**Background:**

This study aimed to evaluate the therapeutic effect of hyperthermic intraperitoneal chemotherapy (HIPEC) in patients with primary ovarian cancer (OC) following cytoreductive surgery (CRS).

**Methods:**

Following the PRISMA (Preferred Reporting Items for Systematic Reviews and Meta-Analyses) guidelines, we searched PubMed, Web of Science, Embase, and the Cochrane Library from inception to May 2025. The outcomes were progression-free survival (PFS) and overall survival (OS). Treatment effects were quantified using pooled hazard ratios (HRs) with 95% confidence intervals (95%CIs).

**Results:**

This meta-analysis included 10 studies [three randomized controlled trials (RCTs) and seven observational studies] involving 1,668 patients. Patients with primary OC undergoing CRS who received HIPEC treatment in the experimental group demonstrated prolonged PFS (HR = 0.45, 95%CI = 0.29–0.69, *p* < 0.001, *I*^2^ = 77.3%) and OS (HR = 0.59, 95%CI = 0.45–0.78, *p* < 0.001, *I*^2^ = 60.6%) outcomes compared with the controls. In the experimental group, better PFS benefits were observed in Western populations, observational studies, interval cytoreductive surgery (ICS), and in patients with Eastern Cooperative Oncology Group (ECOG) 0–1. HIPEC was beneficial for patients with OC regardless of follow-up time ≥5 years (HR = 0.68, 95%CI = 0.49–0.96, *p* = 0.026) or <5 years (HR = 0.29, 95%CI = 0.13–0.63, *p* = 0.002), with a greater magnitude of benefit observed in the subgroup with follow-up <5 years. The subgroup analyses for OS revealed a consistent benefit of HIPEC across Western population, follow-up time ≥5 years, ICS, and ECOG 0–1. Benefit was associated with HIPEC in both RCT (HR = 0.75, 95%CI = 0.60–0.94, *p* = 0.013) and observational analyses (HR = 0.47, 95%CI = 0.33–0.68, *p* = 0.001), while the observed effect size was larger in the latter. Conversely, HIPEC was associated with a significant OS detriment in patients with an ECOG performance status of 2–3 (HR = 2.37, 95%CI = 1.07–5.23, *p* = 0.033).

**Conclusion:**

HIPEC appears beneficial to the prognosis of patients with primary OC following CRS, particularly after ICS. However, the administration of HIPEC in patients with ECOG 2–3 requires careful clinical consideration.

**Systematic Review Registration:**

https://www.crd.york.ac.uk/prospero/, identifier CRD420251036731.

## Introduction

Studies on hyperthermic intraperitoneal chemotherapy (HIPEC) following cytoreductive surgery (CRS) for ovarian cancer (OC) in specific clinical subgroups are still insufficient. The absence of pathognomonic symptoms and effective screening modalities results in the majority of patients presenting with advanced-stage disease, frequently with peritoneal dissemination (1). CRS combined with systemic chemotherapy remains the standard of treatment for OC. A 2019 study reported that, despite aggressive primary treatment with CRS and platinum-based intraperitoneal chemotherapy, the 5-year survival rate was still only 45% (2). Moreover, the treatment compliance rate is low due to complications and systemic toxicity. Among them, approximately 80% of patients develop recurrence requiring additional therapies, with the majority succumbing to the disease within 5 years of diagnosis (3, 4). The combination of CRS and HIPEC for OC was initially implemented for recurrent cancer and was later used for primary cancer (5, 6). Cell experiments have shown that hyperthermia above 42°C triggers tumor cell apoptosis (7). HIPEC induces RNA synthesis arrest, reversible mitotic arrest, changes in the lysosomal enzyme activity, and increased cell membrane permeability in malignant cells (8).

Previous meta-analyses support the potential benefit of HIPEC in OC (9–15). However, trials and subgroup analyses have been limited. Our study strengthens this evidence base by incorporating a more extensive and updated collection of studies. Moreover, subgroup analyses were conducted across clinically relevant factors, including surgery type [i.e., primary cytoreductive surgery (PCS) and interval cytoreductive surgery (ICS)], region, study design, follow-up time, and physical performance status.

## Methods

### Search strategy

This systematic review and meta-analysis adhered to the Preferred Reporting Items for Systematic Reviews and Meta-Analyses (PRISMA) guidelines (16). The review was registered with PROSPERO (ID no. CRD420251036731). PubMed, Web of Science, Embase, and the Cochrane Library were searched from inception to May 2025. The search terms used included MeSH terms such as “Ovarian Neoplasms” and “Hyperthermic Intraperitoneal Chemotherapy,” supplemented with free words such as “Ovarian cancer,” “Ovarian adenocarcinoma,” and “HIPEC.” We also manually screened the references to identify relevant studies. Two researchers independently screened all of the retrieved literature based on predefined criteria, with discrepancies resolved through consensus.

### Inclusion and exclusion criteria

The inclusion criteria were as follows: 1) participants who were diagnosed with primary OC; 2) in the experimental group, patients who received HIPEC after CRS; 3) in the control group, patients who underwent CRS, but did not receive HIPEC; 4) studies that reported the overall survival (OS) or progression-free survival (PFS); and 5) randomized controlled trials (RCTs) or observational studies reported in English.

The exclusion criteria were as follows: 1) studies without available data for analysis; 2) studies with the same participants (only the most recent or comprehensive article was included); and 3) studies with a full text that cannot be obtained.

### Data extraction

Data were independently extracted by two researchers using a predefined form. The extracted information included the authors, publication year, country, International Federation of Gynecology and Obstetrics (FIGO) OC staging, surgery type, number of patients, follow-up time, study design, recruitment period, age, inclusion criteria, and treatment strategy.

### Quality assessment

Two independent reviewers assessed the quality of the observational studies using the Newcastle–Ottawa Scale (NOS). Four points were assigned to the selection criteria, three points to the outcome assessment, and two points to the comparability between groups. Studies were categorized as having low (0–3), moderate (4–6), or high (7–9) quality, with only the high-quality studies included in the analysis.

The quality of the RCTs was evaluated using the Review Manager (RevMan) 5.3 software. Based on the Cochrane Handbook criteria, risk of bias was assessed in aspects such as random sequence generation, allocation concealment, blinding, data integrity, and selective reporting. The “Risk of bias graph” used graphical symbols and colors to visualize the risk of bias of each study across different factors. The “Risk of bias summary” tabulated the evaluation findings. Green represents a “low risk of bias,” yellow represents an “unclear risk of bias,” and red represents a “high risk of bias.” Discrepancies were resolved by a third researcher.

### Statistical analysis

All analyses used Stata 12.0, with two-sided tests and the significance set at *p* < 0.05. The effect of HIPEC on the survival of patients with primary OC after CRS was analyzed using hazard ratios (HRs) and 95% confidence intervals (95%CIs). The HRs and 95%CIs were log-transformed for analysis, with standard errors calculated. Given potential heterogeneity due to the HIPEC protocols, chemotherapeutic agents, patient selection criteria, and age distribution, a random effects model using inverse-variance weighting was employed to pool the data, thereby improving the reliability of the results (17). Heterogeneity among studies was assessed using the chi-square test and the results presented as *I*^2^, with *p* ≤ 0.05 and *I*^2^ ≥ 50% indicating significant heterogeneity. Specifically, *p* > 0.05 and *I*^2^ < 50% indicate low heterogeneity. Publication bias was assessed using Egger’s test. The sensitivity analyses employed the leave-one-out method.

## Results

### Literature search

**Figure 1:**
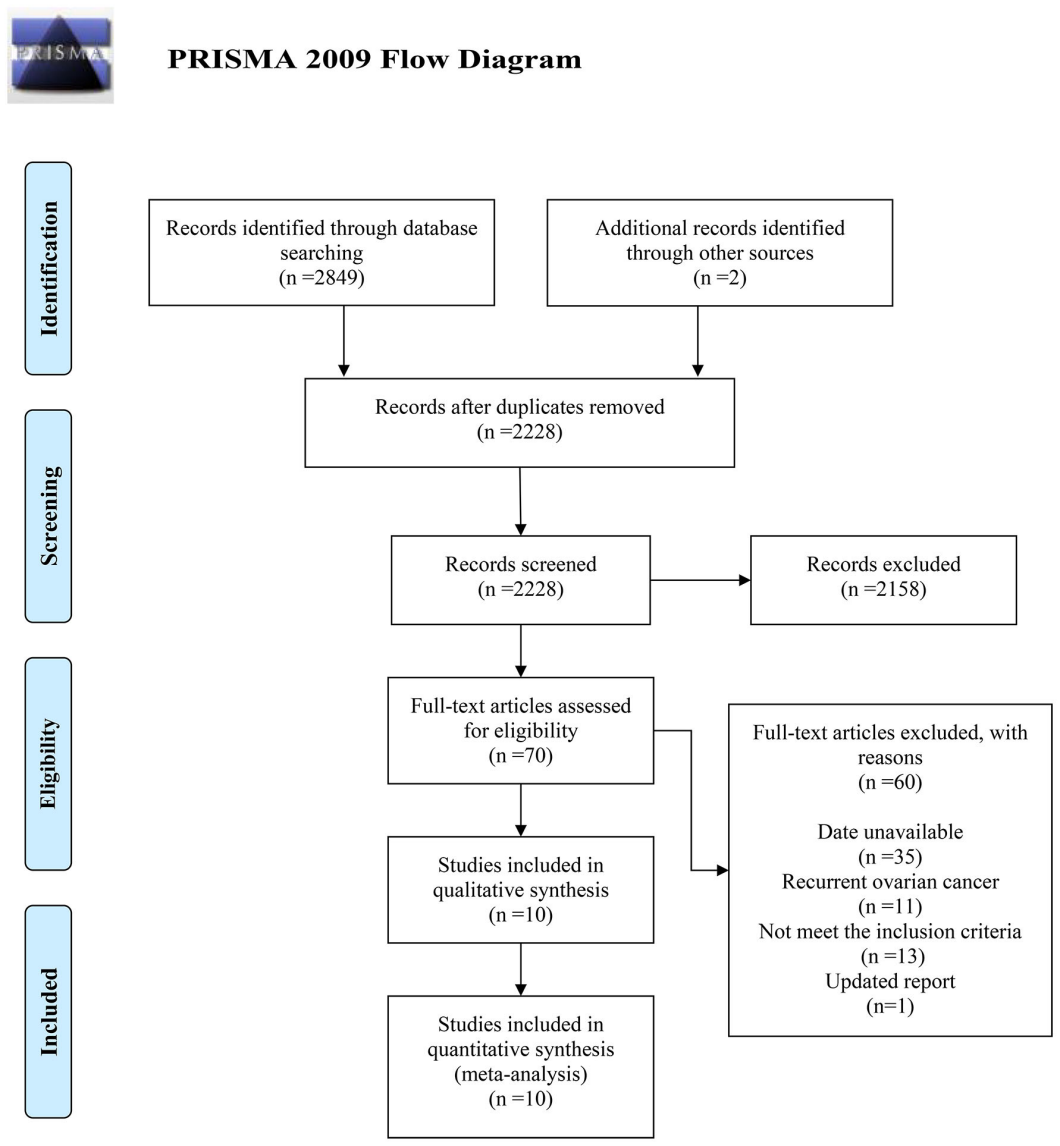
Flow diagram of the selection process.

The search and selection processes are depicted in **Figure 1**. Initially, 2,849 records were retrieved from the electronic databases, supplemented by two additional studies from manual reference screening. After elimination of duplicates, 2,228 articles remained. Based on the screening of the titles and abstracts, 2,158 articles were excluded. Following a full-text review of 70 articles against the selection criteria, 60 were excluded for the following reasons: unavailable data (*n* = 35), recurrent OC (*n* = 11), not meeting the inclusion criteria (*n* = 13), and updated report (*n* = 1). The final analysis included 10 studies (seven observational studies (18–24) and three RCTs (25–27)).

### Study characteristics

The 10 included articles were published between 2005 and 2025. Geographically, three studies were conducted in Eastern countries (China and Korea) and seven in Western countries (Argentina, France, Greece, Netherland, Spain, and USA). Two studies enrolled patients with FIGO stage IC–IIIC, whereas eight studies included those with FIGO stage III–IV. Surgery types consisted of PCS (*n* = 6) and ICS (*n* = 5). A total of 984 patients underwent HIPEC compared with 684 in the control group. Standard HIPEC protocols employed cisplatin, paclitaxel, and carboplatin. Treatment durations ranged from 60 to 120 min at temperatures of 41–43°C. The detailed characteristics of the included studies are presented in **Table 1** and **Supplementary Table S1**.

**Table 1:** Characteristics of the included studies in the meta-analysis.

Author	Year	Country	FIGO staging	Surgery type	No. of HIPEC	No. of non-HIPEC	HIPEC			Follow-up time (months)	Study design
							Medication	Time (min)	Temperature (°C)		
Gori, J.	2005	Argentina	IIIB–IIIC	PCS	32	19	Cisplatin	60	41–43	73	Observational study
Kim, Jin Hwi	2010	Korea	IC–IIIC	PCS	19	24	Paclitaxel	90	43–44	80.2	Observational study
Cascales-Campos, Pedro Antonio	2014	Spain	IIIC–IV	ICS	52	35	Paclitaxel	60	42	36	Observational study
Mendivil, Alberto A.	2017	USA	III–IV	PCS	69	69	Carboplatin	90	41.5	36	Observational study
Antonio, Cascales Campos Pedro	2021	Spain	IIIB–IIIC	ICS	35	36	Cisplatin	60	42–43.8	32	RCT
Lim, Myong Cheol	2022	Korea	III–IV	PCS, ICS	92	92	Cisplatin	90	41.5	69.4	RCT
Aronson, S. L.	2023	Netherlands	III	ICS	122	123	Cisplatin	90	40–42	121	RCT
Frankinet, Lisa	2023	France	IIIC–IVA	ICS	59	55	Cisplatin, oxaliplatin	90	41–43	60	Observational study
Karanikas, Michail	2024	Greece	IC–IIIC	PCS	79	72	Cisplatin	90	42–43	22	Observational study
Lei, Ziying	2025	China	III	PCS	425	159	Cisplatin	60	43	87.2	Observational study

RCT, randomized controlled trial; PCS, primary cytoreductive surgery; NA, not available; ICS, interval cytoreductive surgery; HIPEC, hyperthermic intraperitoneal chemotherapy; FIGO, The International Federation of Gynecology and Obstetrics.

### Bias risk assessment

The NOS was employed to objectively assess the quality of the included observational studies. The results revealed that three studies scored 9 points, three scored 8 points, and one scored 7 points. The detailed bias risk assessments are documented in **Supplementary Table S2**, with scoring reasons providing details of the NOS scores.

The RevMan 5.3 software was applied for quality assessment of the RCTs. The overall risk of bias in the “risk of bias graph” was low (**Supplementary Figure S1**), with scoring reasons provided in the “Details of risk of bias score.” High risk was limited to “performance bias” and “other bias.” Unclear risk was limited to “detection bias” and “other bias.” In the “Risk of bias summary” (**Supplementary Figure S2**), individual study assessments showed: Antonio et al. to have high risk of “performance bias” and “other bias” and an unclear risk of “detection bias”; Lim et al. showed an unclear risk in “other bias”; and Aronson et al. had a low risk in all domains.

### Progression-free survival

**Figure 2:**
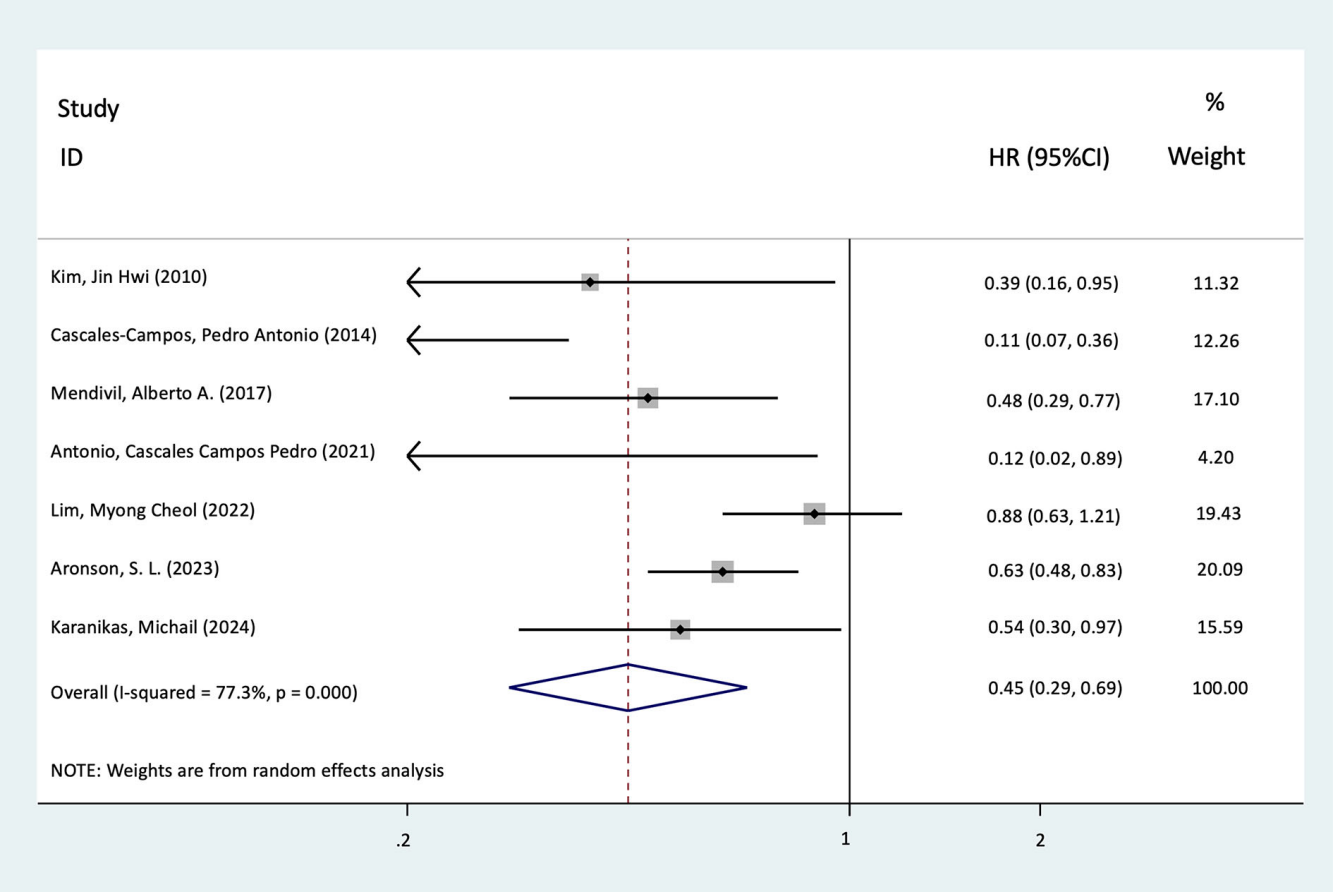
Forest plot of the meta-analysis evaluating the effect of hyperthermic intraperitoneal chemotherapy (HIPEC) on progression-free survival (PFS) in primary ovarian cancer (OC) patients following cytoreductive surgery (CRS) (*p* < 0.001).

There were seven studies that reported the PFS with HRs and 95%CIs. A total of 468 patients received HIPEC compared with 451 patients in the control group. Patients who received HIPEC after CRS for primary OC showed significantly better PFS relative to the control group (HR = 0.45, 95%CI = 0.29–0.69, *p* < 0.001, *I*^2^ = 77.3%). Further details are presented in **Figure 2**.

Two Eastern studies reported the PFS and showed no significant difference between the HIPEC and non-HIPEC groups (HR = 0.65, 95%CI = 0.30–1.41, *p* = 0.278). Conversely, five studies in Western countries reported the PFS, demonstrating that patients in the HIPEC group had superior PFS compared with the controls (HR = 0.37, 95%CI = 0.21–0.65, *p* = 0.001). In the subgroup analysis of four observational studies, the patients in the HIPEC group exhibited superior PFS compared with the control group (HR = 0.34, 95%CI = 0.18–0.65, *p* = 0.001). Conversely, in the subgroup comprising three RCTs, there was no significant difference between the two groups in PFS (HR = 0.67, 95%CI = 0.43–1.05, *p* = 0.084). In the subgroup analyses, patients who received HIPEC demonstrated a superior PFS compared with those in the non-HIPEC group, regardless of whether the follow-up time was <5 years (HR = 0.29, 95%CI = 0.13–0.63, *p* = 0.002) or ≥5 years (HR = 0.68, 95%CI = 0.49–0.96, *p* = 0.026). The surgery types were categorized as PCS and ICS based on pre-surgical treatments. There was no statistically significant difference in the PFS between the two groups of patients undergoing PCS (HR = 0.62, 95%CI = 0.37–1.03, *p* = 0.063). In the ICS subgroup, HIPEC patients demonstrated better PFS outcomes than patients in the control group (HR = 0.33, 95%CI = 0.15–0.72, *p* = 0.005). Among the patients with Eastern Cooperative Oncology Group (ECOG) performance status 0–1, those who received HIPEC demonstrated better improvement in PFS compared with the patients in the control group (HR = 0.33, 95%CI = 0.13–0.83, *p* = 0.019). Details of the subgroup analysis are presented in **Table 2**.

**Table 2:** Subgroup analyses assessing the association between hyperthermic intraperitoneal chemotherapy (HIPEC) and progression-free survival (PFS).

Subgroup	No. of research	No. of HIPEC	No. of non-HIPEC	HR	95%CI	*p*	*I*^2^ (%)
Region							
East	2	111	116	0.65	0.30–1.41	0.278	64.6
West	5	357	335	0.37	0.21–0.65	0.001	77.9
Study design							
Observational study	4	219	200	0.34	0.18–0.65	0.001	72.9
RCTs	3	249	251	0.67	0.43–1.05	0.084	65.3
Follow-up time							
<5 years	4	235	212	0.29	0.13–0.63	0.002	75.9
≥5 years	3	233	239	0.68	0.49–0.96	0.026	51.4
Surgery type							
PCS	4	225	214	0.62	0.37–1.03	0.063	68.0
ICS	4	243	237	0.33	0.15–0.72	0.005	83.6
ECOG							
0–1	4	248	232	0.33	0.13–0.83	0.019	88.2

RCTs, randomized controlled trials; PCS, primary cytoreductive surgery; ICS, interval cytoreductive surgery; HR, hazard ratio; ECOG, Eastern Cooperative Oncology Group; 95%CI, 95% confidence interval.

### Overall survival

**Figure 3:**
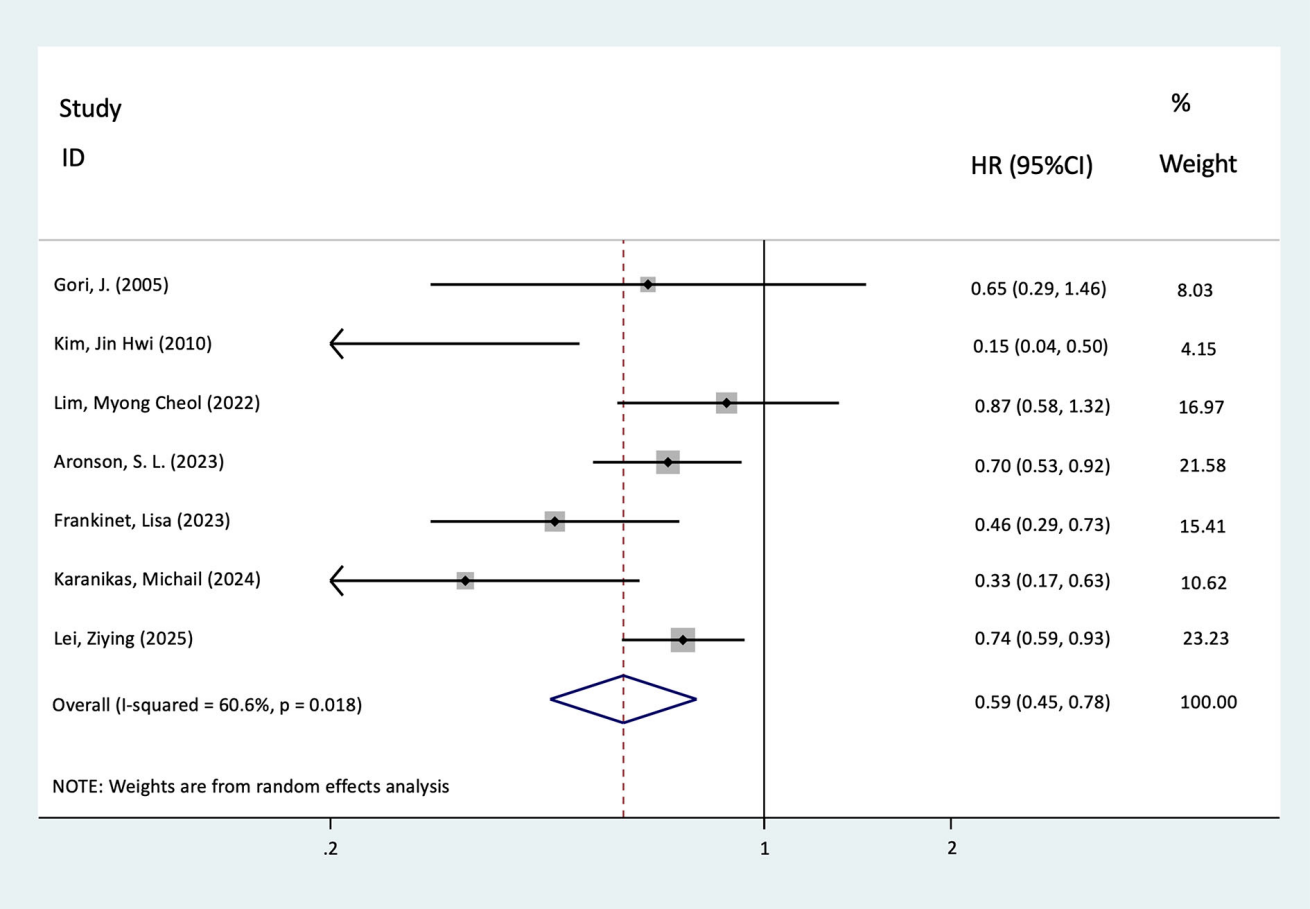
Forest plot of the meta-analysis evaluating the effect of hyperthermic intraperitoneal chemotherapy (HIPEC) on overall survival (OS) in primary ovarian cancer (OC) patients following cytoreductive surgery (CRS) (*p* < 0.001).

There were seven studies involving 828 HIPEC-treated patients and 544 controls that reported the OS. Pooled analysis showed that patients who received HIPEC had better OS outcomes compared with those who did not receive HIPEC (HR = 0.59, 95%CI = 0.45–0.78, *p* < 0.001, *I*^2^ = 60.6%). **Figure 3** provides a detailed breakdown of these findings.

In the region subgroup analyses, HIPEC was associated with improved OS in Western populations (HR = 0.54, 95%CI = 0.38–0.76, *p* < 0.001), while a non-significant benefit was observed in Eastern populations (HR = 0.65, 95%CI = 0.39–1.07, *p* = 0.092). In the observational study (HR = 0.47, 95%CI = 0.33–0.68, *p* = 0.001) and RCT subgroups (HR = 0.75, 95%CI = 0.60–0.94, *p* = 0.013), patients who received HIPEC had better OS than those in the control groups in both cases. Notably, HIPEC showed superior benefits in observational studies than in RCTs. For the subgroup with ≥5 years of follow-up, patients who received HIPEC had better OS outcomes than those in the control group (HR = 0.65, 95%CI = 0.50–0.83, *p* = 0.001). HIPEC significantly improved the OS in the ICS subgroup (HR = 0.59, 95%CI = 0.45–0.78, *p* < 0.001), but not in the PCS subgroup (HR = 0.58, 95%CI = 0.34–1.00, *p* = 0.052), a finding consistent with the PFS results. In the physical performance status subgroup, HIPEC demonstrated superior OS benefits specifically for patients with ECOG 0–1 (HR = 0.76, 95%CI = 0.62–0.93, *p* = 0.008). Conversely, among those with ECOG 2–3, HIPEC patients showed inferior OS outcomes compared with those in the control group (HR = 2.37, 95%CI = 1.07–5.23, *p* = 0.033). Additional information is provided in **Table 3**.

**Table 3:** Subgroup analyses assessing the association between hyperthermic intraperitoneal chemotherapy (HIPEC) and overall survival (OS).

Subgroup	No. of research	No. of HIPEC	No. of non-HIPEC	HR	95%CI	*p*	*I*^2^ (%)
Region							
East	3	536	275	0.65	0.39–1.07	0.092	70.39
West	4	292	269	0.54	0.38–0.76	<0.001	47.1
Study design							
Observational study	5	614	329	0.47	0.33–0.68	0.001	53.4
RCTs	2	214	215	0.75	0.60–0.94	0.013	0
Follow-up time							
≥5 years	6	698	429	0.65	0.50–0.83	0.001	53.0
Surgery type							
PCS	5	613	323	0.58	0.34–1.00	0.052	74.7
ICS	3	215	221	0.59	0.45–0.78	<0.001	23.2
ECOG							
0–1	2	379	209	0.76	0.62–0.93	0.008	0
2–3	2	84	74	2.37	1.07–5.23	0.033	29.2

RCTs, randomized controlled trials; PCS, primary cytoreductive surgery; ICS, interval cytoreductive surgery; HR, hazard ratio; ECOG, Eastern Cooperative Oncology Group; 95%CI, 95% confidence interval.

### Publication bias and sensitivity analysis

The publication bias of the pooled results on the effect of HIPEC for primary OC patients after CRS on PFS was assessed. The results of Egger’s test revealed no significant publication bias (*p* = 0.072). The relevant details are shown in **Supplementary Figure S3**. Sensitivity analysis was conducted using the leave-one-out method, which demonstrated the stability of the statistical results. Detailed results are presented in **Supplementary Figure S4**. Publication bias in the pooled results for the effect of HIPEC for primary OC patients after CRS on OS was evaluated. No significant publication bias was detected (*p* = 0.072). Details can be found in **Supplementary Figure S5**. The sensitivity analysis indicated stable results. **Supplementary Figure S6** presents detailed information.

## Discussion

This meta-analysis aimed to investigate the effect of HIPEC on the survival rates of patients with primary OC after CRS. The study included 10 articles, consisting of three RCTs and seven observational studies involving more than 1,600 patients. The results indicated that HIPEC significantly improved the survival outcomes of patients with primary OC after CRS. Specifically, HIPEC improved the PFS by 55% and the OS by 41%. The survival benefit of HIPEC was primarily confined to Western populations and to patients who received ICS. However, the magnitude of this benefit appears to be overestimated in observational studies and tends to diminish with longer follow-up. Critically, the efficacy of HIPEC is dependent on the performance status, providing a significant survival advantage for patients with ECOG 0–1, but potentially harming those with ECOG 2–3.

OC is a gynecological malignancy with an extremely high recurrence rate, predominantly presenting as peritoneal metastasis. Anatomically, the pelvic and abdominal cavities communicate via structures including the fallopian tubes and the ovarian ligaments, providing a pathway for cancer cell dissemination (28). The peritoneum, which is enriched with vascular and lymphatic networks, facilitates cancer cell implantation and metastasis. Peritoneal fluid dynamics contribute to the widespread intraperitoneal dissemination of malignant cells (29). Cellular interactions between neoplastic cells and cancer-associated fibroblasts generate tumor-associated fibrosis and the formation of an immunosuppressive microenvironment. These processes promote angiogenesis by altering collagen expression, increasing matrix stiffness, and inducing a highly oriented collagen fiber remodeling, thereby supporting tumor cell survival (30, 31).

HIPEC was developed based on the aforementioned theory of peritoneal metastasis. While both conventional intraperitoneal chemotherapy and HIPEC deliver cytotoxic agents directly into the peritoneal cavity, HIPEC distinguishes itself by the synergistic combination of hyperthermia and chemotherapy (32). This integrated approach leverages the peritoneal–plasma barrier to maintain high intraperitoneal drug concentrations while limiting systemic exposure. The technique delays drug diffusion into the systemic circulation and reduces intraperitoneal clearance. Furthermore, drugs within the peritoneal cavity are absorbed via the portal vein system, thereby increasing cytotoxic exposure to potential hepatic micrometastases (33). From the perspective of thermal effects, an increased tumor blood flow during treatment represents the most critical physiological response (34). For instance, blood vessels with a diameter of 40 μm can dilate to 95 μm at 42°C, and the red blood cell flow velocity can triple in some vessels (35). Hyperthermia enhances the drug accumulation in tumor tissues by improving blood perfusion. At the same time, the hyperthermic environment alters the permeability of the tumor cell membranes and nuclear membranes to drugs, induces protein denaturation, and inhibits DNA repair function (8). As normal tissues generally exhibit higher thermal tolerance than malignant cells, HIPEC selectively targets tumor cells while limiting damage to healthy tissues, thereby improving the therapeutic index (36). The combination of hyperthermia and chemotherapy produces a synergistic effect, significantly enhancing therapeutic outcomes.

In the analyses of both PFS and OS, HIPEC demonstrated survival benefits for patients with OC from Western countries, whereas no significant benefit was observed in patients from Eastern countries. Potential regional factors contributing to this discrepancy may include genetic and ethnic variations in the drug-metabolizing enzymes and the DNA repair pathways, which could influence both the chemosensitivity and the treatment toxicity during HIPEC. A study on HIPEC for appendiceal cancer has shown that non-Hispanic black patients have lower survival rates than Hispanics. Eastern country populations may exhibit lower tolerance to hyperthermia and chemotherapy, leading to the attenuation of the therapeutic efficacy of HIPEC (37). It should also be noted that the limited sample size from Eastern countries (comprising only China and Korea) reduced the statistical power of this subgroup analysis.

Observational studies inherently carry lower credibility than RCTs. Our findings indicate that observational studies tend to overestimate the therapeutic efficacy of HIPEC, potentially due to researchers unconsciously enrolling patients with more favorable characteristics into the HIPEC group. Patients in the experimental group may have had better socioeconomic status, better treatment adherence, and enhanced physiological resilience to tolerate prolonged therapy and complications. These inherent health advantages can independently improve the survival outcomes, thereby inflating the perceived benefit of HIPEC. In contrast, RCTs mitigate such bias through a balanced allocation, providing more reliable efficacy estimates.

This study found that the survival benefit of HIPEC was more pronounced during a follow-up time of <5 years, with its beneficial effect diminishing with a follow-up time ≥5 years. The recurrence preventive effect of HIPEC is limited, exhibiting reduced efficacy against resistant tumor cells responsible for late recurrences. Furthermore, differences in the subsequent treatments received by the patients in both groups during long-term follow-up may also confound the independent treatment effect of HIPEC (38). In the initial period, the mortality risk predominantly stems from the cancer itself, allowing the direct antitumor effects of HIPEC, such as the eradication of minimal residual disease and the suppression of peritoneal metastasis. However, with time, the mortality risk from age-related conditions, such as cardiovascular disease and organ failure, becomes increasingly prominent. These non-oncological factors weaken the statistical survival advantage attributable to HIPEC. These findings suggest that the principal value of HIPEC lies in providing a crucial early survival advantage, whereas long-term outcomes are increasingly influenced by a patient’s overall health status and comprehensive treatment strategies.

Compared with those with PCS, patients undergoing ICS derive greater benefit from HIPEC. Biologically, neoadjuvant chemotherapy induces tumor cell apoptosis and disrupts the DNA repair mechanisms, thereby enhancing the susceptibility of residual tumor cells to hyperthermia and intraperitoneal chemotherapy. Furthermore, it reduces the tumor burden, reducing the risk of postoperative recurrence (39, 40). Because no macroscopic residual disease or minimal residual disease is a prerequisite for HIPEC to effectively eradicate microscopic residual disease, this combined biological and surgical optimization creates an ideal setting for HIPEC to achieve maximal efficacy, ultimately amplifying the therapeutic benefit in ICS patients.

A patient’s performance status is a critical determinant of HIPEC outcomes. Our meta-analysis revealed that HIPEC provides a survival advantage for patients with ECOG 0–1. In contrast, it is associated with worse OS in patients with ECOG 2–3. Patients with ECOG 2–3 often have multi-organ dysfunction. They may not be able to tolerate the circulatory stress from hyperthermia or the chemotherapeutic toxicity to the liver and kidneys during HIPEC. Slowed drug metabolism leads to drug accumulation in the body, increasing the risk of drug toxicity (41). Patients with a high ECOG score may have malnutrition and low immune function due to tumor consumption and decreased appetite. These conditions impair the treatment response and the tissue repair capacity. In addition, patients with higher ECOG scores may have limited ability to engage in rehabilitation due to discomfort and functional limitations, leading to a delayed physical recovery and compromising the treatment outcomes.

This meta-analysis focused on high-quality RCTs and observational studies and included only patients with primary OC. HR was used as the outcome indicator for the evaluation of the PFS and OS of patients. This meta-analysis included the most comprehensive original articles to date within the HIPEC research domain for primary OC. Compared with the meta-analysis by Filis et al. (10), which included both primary and recurrent OC with a total of 737 participants, our study focused exclusively on primary disease and encompassed 1,668 participants. Compared with the meta-analysis published by Taliento et al. (13), we have additionally conducted subgroup analyses on region, follow-up time, and ECOG, among others. This study stratified populations to determine which patients benefited from HIPEC, thereby enhancing its clinical applicability.

### Limitation

However, this study has several limitations. The overall number of included RCTs was limited, and the sample sizes for certain subgroup analyses were small, which may have increased the risk of statistical effect and compromised the stability of the findings. Furthermore, the heterogeneity in the HIPEC protocols (e.g., the choice of chemotherapeutic agents, duration, and temperature) presented a challenge for drawing unified conclusions. The inclusion of observational studies with their inherent retrospective design introduced potential biases. The lack of detailed data, such as details on age and histological subtypes, limited a more comprehensive subgroup analyses. Finally, the potential for publication bias must be acknowledged as the limited number of studies reduced the statistical power to reliably detect this.

## Conclusion

HIPEC conveys a prognostic benefit in primary OC, with the benefit being most pronounced in Western populations and following ICS. However, the magnitude of this survival advantage appears to diminish with longer follow-up time. Furthermore, the treatment efficacy is highly dependent on a patient’s performance status, offering significant benefit to those with ECOG 0–1, but potentially harming those with ECOG 2–3. Consequently, the application of HIPEC requires careful patient selection. Larger-scale RCTs are required to validate these findings.

## Supplementary material

Supplementary Figure 1 Risk of bias graph for included RCTs.

Supplementary Figure 2 Risk of bias summary for included RCTs.

Supplementary Figure 3 Egger’s test results for publication bias in the meta-analysis of the effect of HIPEC on PFS in primary OC patients following CRS (P = 0.072).

Supplementary Figure 4 Sensitivity analysis of the meta-analysis of the effect of HIPEC on PFS in primary OC patients following CRS.

Supplementary Figure 5 Egger’s test results for publication bias in the meta-analysis of the effect of HIPEC on OS in primary OC patients following CRS (P = 0.072).

Supplementary Figure 6 Sensitivity analysis of the meta-analysis of the effect of HIPEC on OS in primary OC patients following CRS.

Supplementary Table 1 Characteristics of included studies in the meta-analysis.

Supplementary Table 2 Quality assessment of observational studies included.
