# Supplementary figures and images for: Hyperthermic intraperitoneal chemotherapy enhances survival outcomes in primary ovarian cancer following cytoreductive surgery: a systematic review and meta-analysis
Source: Front Oncol. 2025 Dec 3;15:1708318. doi: 10.3389/fonc.2025.1708318 (PMC12709118; doi:10.3389/fonc.2025.1708318)

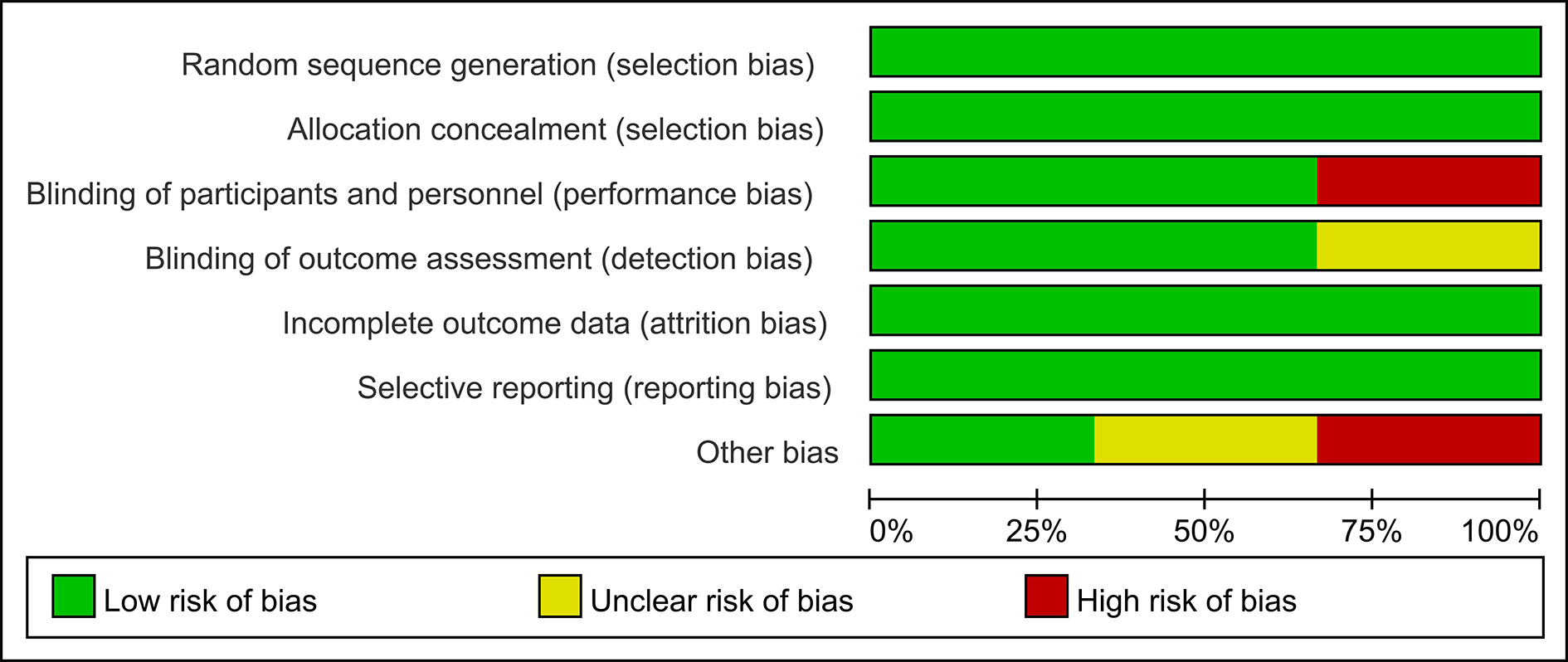

Supplement: Supplementary Figure 1 — Risk of bias graph for included RCTs. [file Image1.tif]

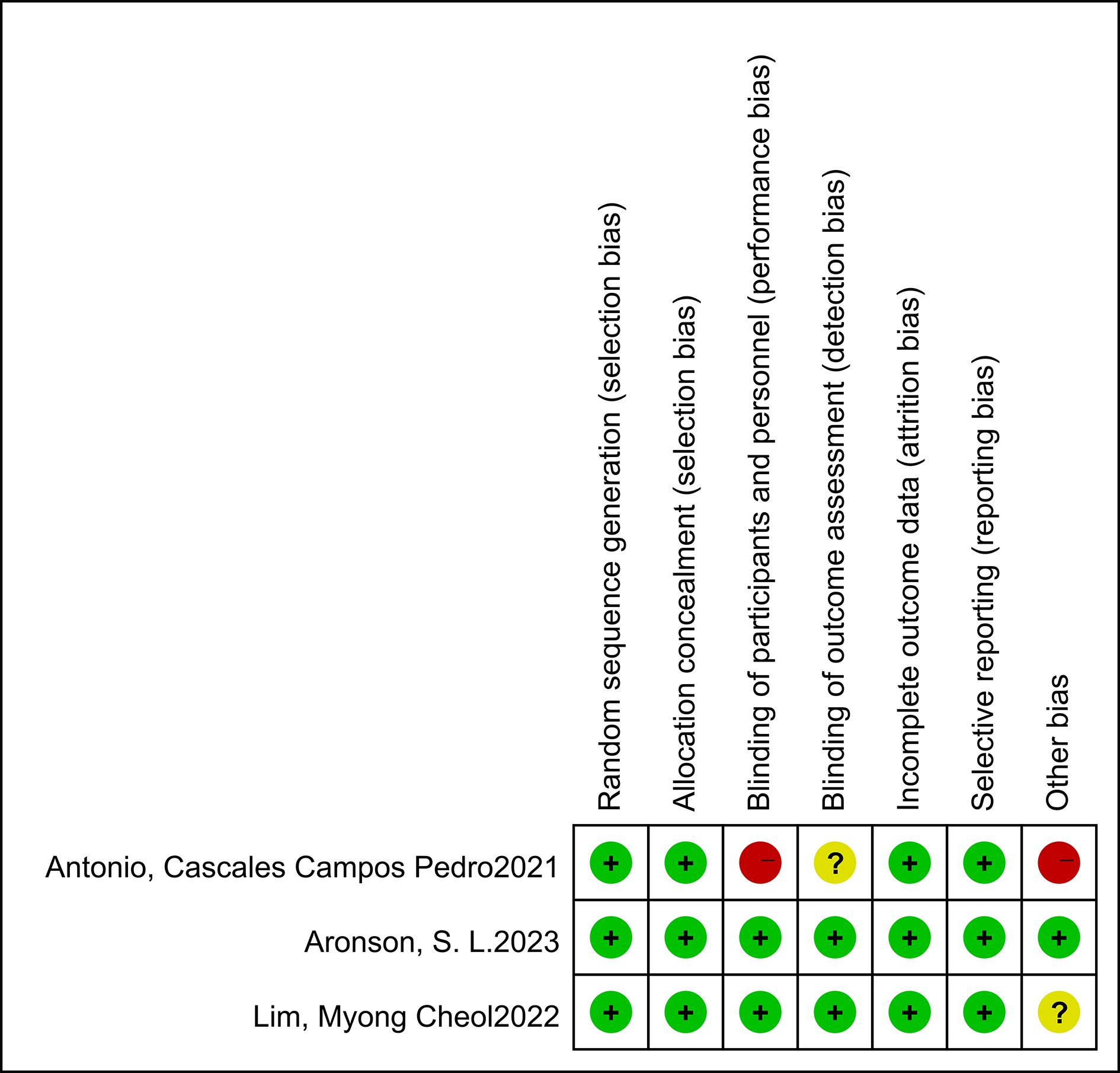

Supplement: Supplementary Figure 2 — Risk of bias summary for included RCTs. [file Image2.tif]

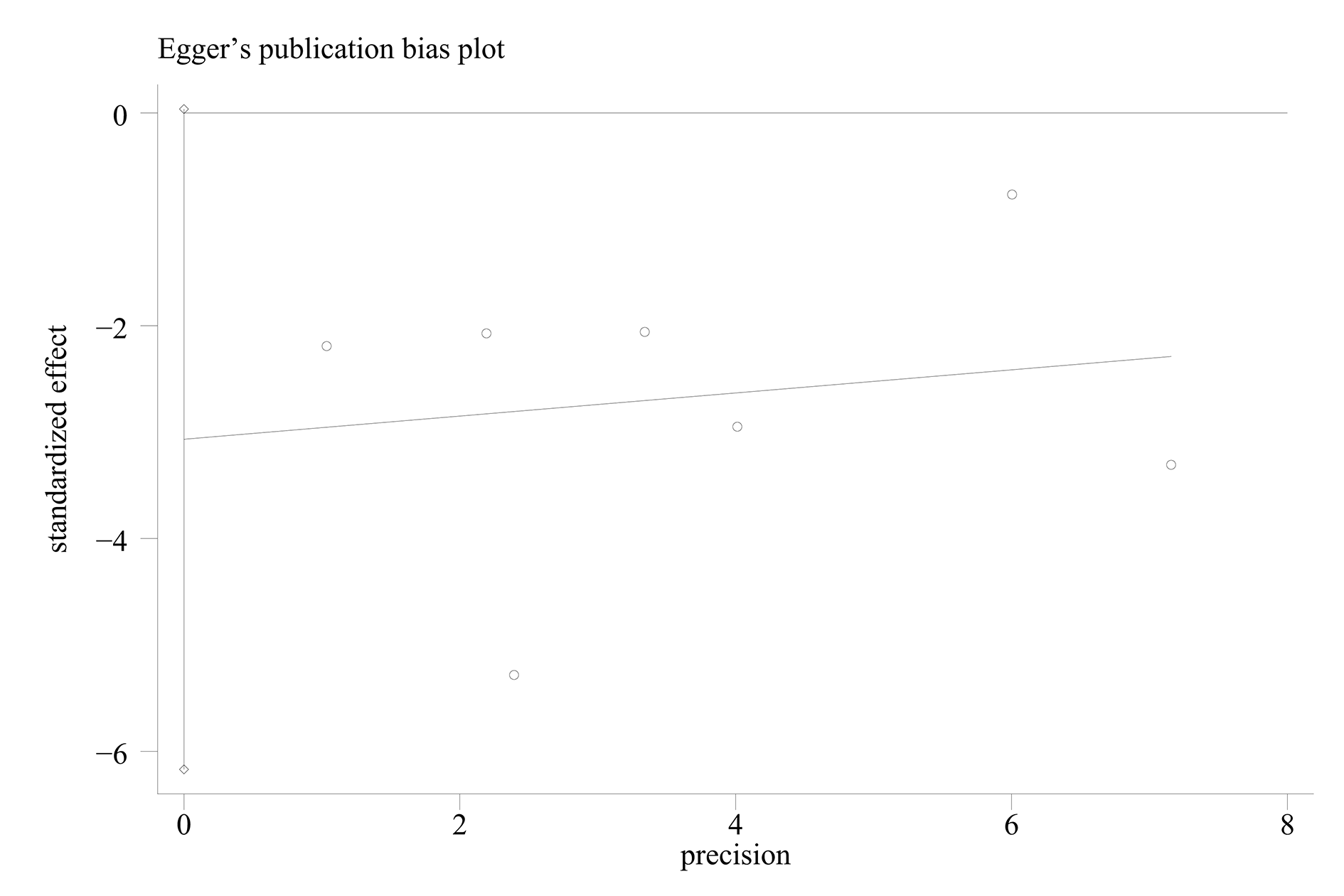

Supplement: Supplementary Figure 3 — Egger’s test results for publication bias in the meta-analysis of the effect of HIPEC on PFS in primary OC patients following CRS (P = 0.072). [file Image3.tif]

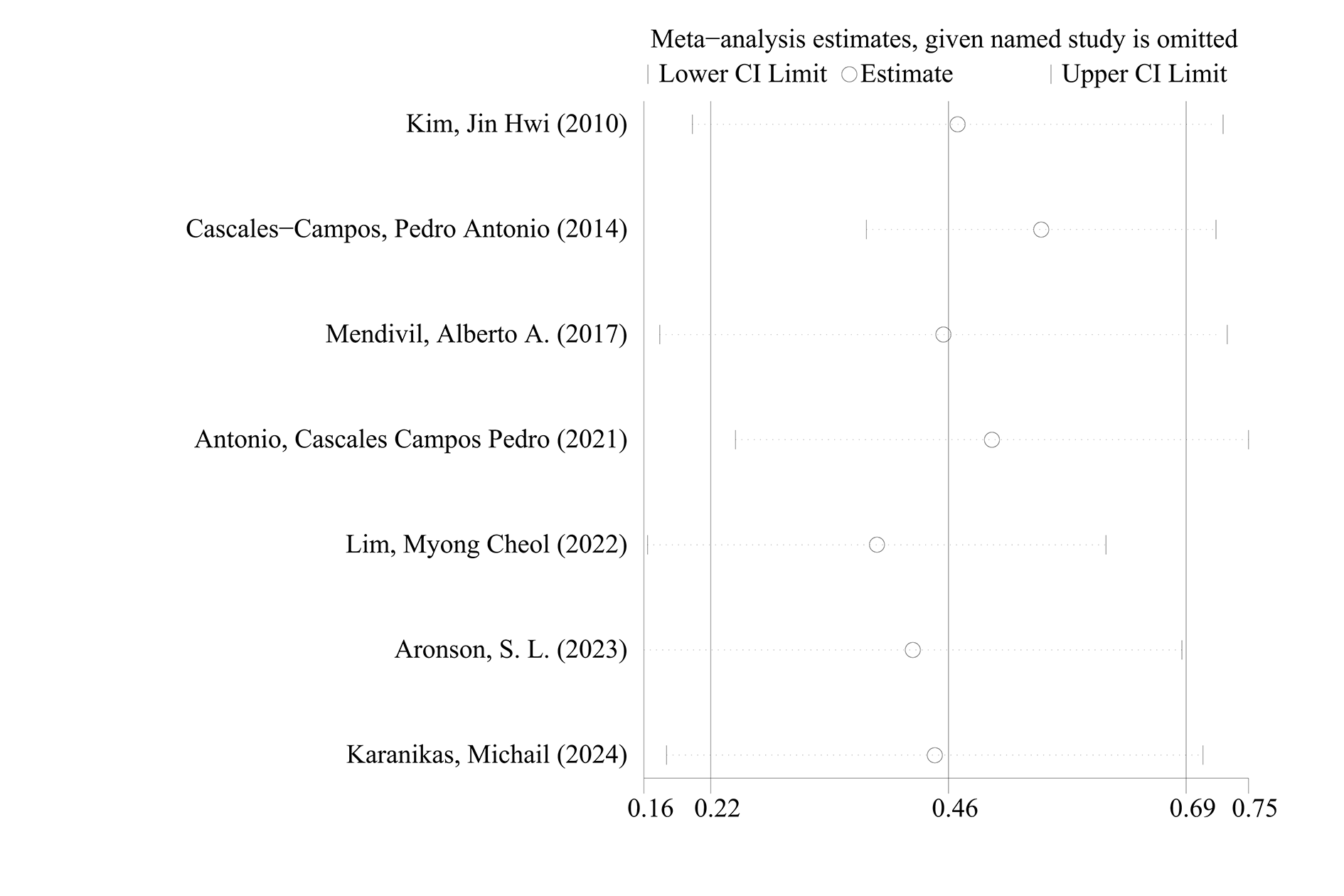

Supplement: Supplementary Figure 4 — Sensitivity analysis of the meta-analysis of the effect of HIPEC on PFS in primary OC patients following CRS. [file Image4.tif]

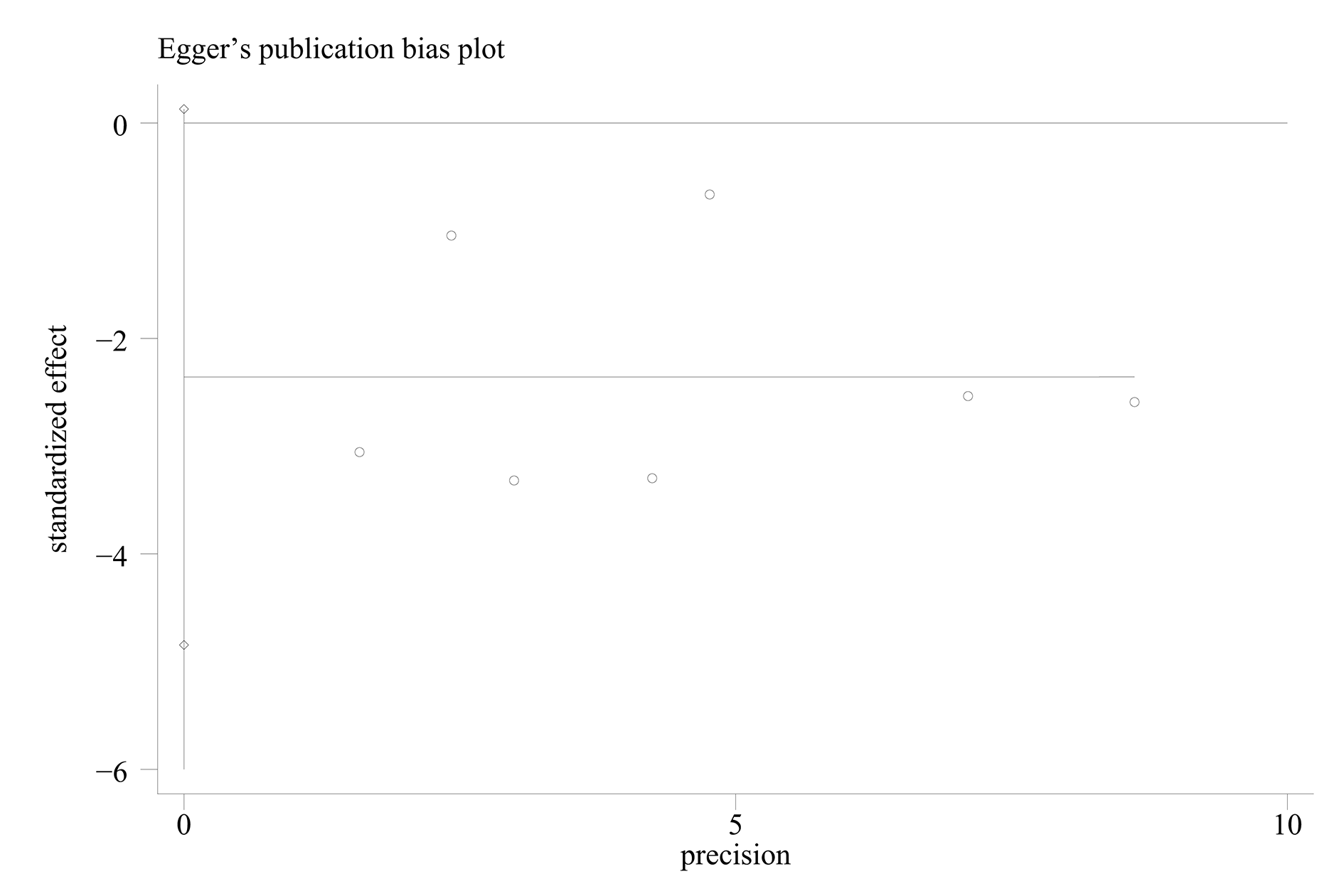

Supplement: Supplementary Figure 5 — Egger’s test results for publication bias in the meta-analysis of the effect of HIPEC on OS in primary OC patients following CRS (P = 0.072). [file Image5.tif]

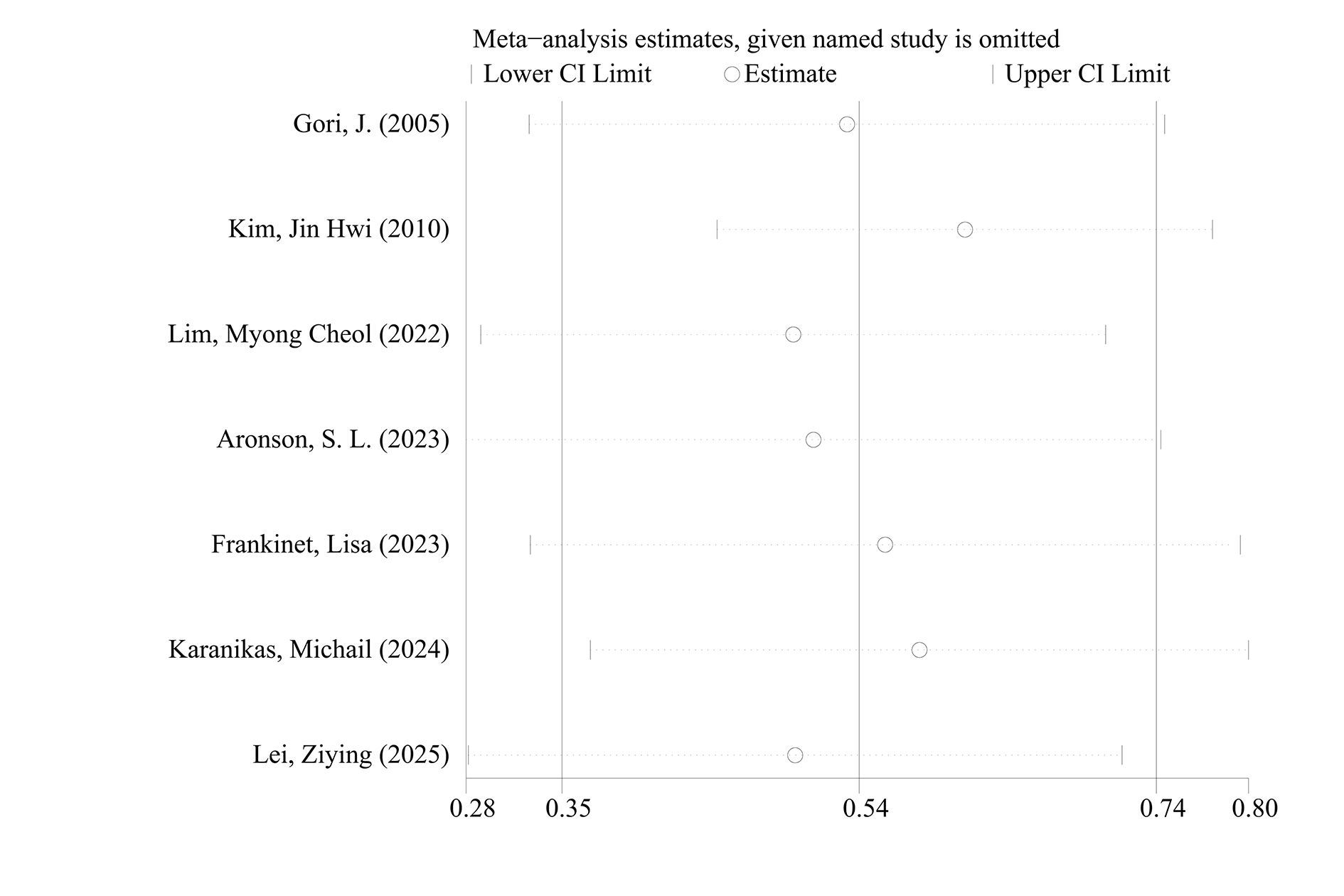

Supplement: Supplementary Figure 6 — Sensitivity analysis of the meta-analysis of the effect of HIPEC on OS in primary OC patients following CRS. [file Image6.tif]
