# Supplementary material for: Hyperthermic intraperitoneal chemotherapy enhances survival outcomes in primary ovarian cancer following cytoreductive surgery: a systematic review and meta-analysis
Source: Front Oncol. 2025 Dec 3;15:1708318. doi: 10.3389/fonc.2025.1708318 (PMC12709118; doi:10.3389/fonc.2025.1708318)
Supplement: Supplementary Table 1 — Characteristics of included studies in the meta-analysis. [file Table1.docx]

**Supplementary Table 1. Characteristics of included studies in the meta-analysis.**

| Author | Year | Source of population | Recruitment period | HIPEC group mean age | Non-HIPEC group mean age | Inclusion criteria | Control group treatment strategy |
| --- | --- | --- | --- | --- | --- | --- | --- |
| Gori, J. | 2005 | Argentine gynecological services | 1991-1997 | 57 | 58 | ovarian epithelial  carcinoma stages IIIB and IIIC, operated between January 1991 and December 1997 | primary cytoreductive surgery (PCS), 6 cycles of intravenous chemotherapy |
| Kim, Jin Hwi | 2010 | Kangnam St. Mary’s Hospital in Seoul | 1999-2004 | 48 | 49 | ≤75 years, confirmed diagnosis, Gynecologic Oncology Group (GOG)0-2; adequate cardiac, renal, hepatic, and bone marrow function, no evidence of residual lesions | PCS, intravenous chemotherapy |
| Cascales-Campos, Pedro Antonio | 2014 | Virgen De La Arrixaca University Hospital, Murcia, Spain | 1998-2011 | 57 | 57 | patients operated on after being diagnosed of stage IIIC/IV ovarian carcinoma | 6–8 cycles of a combination of platinum and taxanes, interval cytoreductive surgery (ICS) |
| Mendivil, Alberto A. | 2017 | Gynecologic Oncology Associates | 2012-2015 | 59.8 | 62.9 | adequate bone marrow, renal, hepatic function and blood coagulation parameters, Eastern Cooperative Oncology Group (ECOG) 0–2 | PCS, 6 cycles of intravenous paclitaxel and carboplatin |
| Antonio, Cascales Campos Pedro | 2021 | Virgen de la Arrixaca University Hospital | 2012-2018 | 56 | 65.5 | ≤75 years, ECOG 0–1, adequate systemic function, consented to participate in the study. | 3 cycles of systemic NACT with carboplatin and paclitaxel, ICS |
| Lim, Myong Cheol | 2022 | National Cancer Center and Ajou University Hospital | 2010-2016 | 52 | 53.5 | ≤75 years, diagnosed advanced (stage III or IV) epithelial ovarian, primary peritoneal, or fallopian tube cancer, ECOG 0-1 | 3 cycles of NACT with carboplatin and paclitaxel, ICS, 6 cycles of intravenous paclitaxel and carboplatin |
| Aronson, S. L. | 2023 | Netherlands Comprehensive Cancer Organisation | 2007-2016 | 63 | 61 | newly diagnosed stage III epithelial ovarian, fallopian tube, or peritoneal cancer | 3 cycles of neoadjuvant chemotherapy (NACT) with carboplatin and paclitaxel, ICS |
| Frankinet, Lisa | 2023 | French Oncologic Gynecologic  hyperthermic intraperitoneal chemotherapy (HIPEC) | 2005-2015 | NA | NA | undergoing cytoreductive surgery (CRS) in first line from 2005 to 2015 | 3-4 cycles Neoadjuvant systemic chemotherapy, ICS, 6 cycles of chemotherapy |
| Karanikas, Michail | 2024 | NA | 2000-2020 | NA | NA | diagnosed ovarian cancer from 2000 until 2020 | PCS, adjuvant chemotherapy with platinum and taxanes derivatives |
| Lei, Ziying | 2025 | Chinese Peritoneal Oncology Study group | 2010-2017 | 55.1 | 54.6 | stage III primary epithelial ovarian, confirmed diagnosis of epithelial ovarian cancer, no antitumor treatment before the operation, no evidence of extra-abdominal metastasis | PCS, intravenous chemotherapy |

PCS: primary cytoreductive surgery; NACT: neoadjuvant chemotherapy; NA: not available; ICS: interval cytoreductive surgery; HIPEC: hyperthermic intraperitoneal chemotherapy; GOG: Gynecologic Oncology Group; ECOG: Eastern Cooperative Oncology Group; CRS: cytoreductive surgery
