# Supplementary material for: Hyperthermic intraperitoneal chemotherapy enhances survival outcomes in primary ovarian cancer following cytoreductive surgery: a systematic review and meta-analysis
Source: Front Oncol. 2025 Dec 3;15:1708318. doi: 10.3389/fonc.2025.1708318 (PMC12709118; doi:10.3389/fonc.2025.1708318)
Supplement: Supplementary Table 2 — Quality assessment of observational studies included. [file Table2.docx]

**Supplementary Table 2.** **Quality assessment of observational studies included.**

| Author | year | **Selection (Out of 4)** | | | | **Comparability**  **(Out of 2)** | **Outcomes (Out of 3)** | | | **Total**  **(Out of 9)** |
| --- | --- | --- | --- | --- | --- | --- | --- | --- | --- | --- |
|  |  | Adequate case definition | Representativeness of the cases | Selection of controls | Definition of controls |  | Ascertainment of exposure | Same method of ascertainment for cases and controls | Non-response rate |  |
| Gori, J. | 2005 | 1 | 1 | 1 | 1 | 2 | 1 | 1 | 1 | 9 |
| Kim, Jin Hwi. | 2010 | 1 | 1 | 1 | 0 | 2 | 1 | 1 | 1 | 8 |
| Cascales-Campos, Pedro Antonio. | 2014 | 1 | 1 | 0 | 1 | 2 | 1 | 1 | 1 | 8 |
| Mendivil, Alberto A. | 2017 | 1 | 1 | 1 | 1 | 2 | 1 | 1 | 1 | 9 |
| Frankinet, Lisa. | 2023 | 1 | 1 | 1 | 1 | 1 | 1 | 1 | 1 | 9 |
| Karanikas, Michail. | 2024 | 1 | 1 | 0 | 0 | 2 | 1 | 1 | 1 | 7 |
| Lei, Ziying. | 2025 | 1 | 1 | 1 | 0 | 2 | 1 | 1 | 1 | 8 |

The observational studies were assessed by the Newcastle-Ottawa Quality Assessment Scale (NOS) checklist.
