# Supplementary material for: Hyperthermic intraperitoneal chemotherapy enhances survival outcomes in primary ovarian cancer following cytoreductive surgery: a systematic review and meta-analysis
Source: Front Oncol. 2025 Dec 3;15:1708318. doi: 10.3389/fonc.2025.1708318 (PMC12709118; doi:10.3389/fonc.2025.1708318)
Supplement: Supplementary file 9 [file Table3.docx]

Details of NOS score.

| Author, year | score | Reasons |
| --- | --- | --- |
| Gori, J. 2005 | Adequate case definition: 1 | The study included 51 patients with ovarian epithelial carcinoma stages IIIB and IIIC who were operated between January 1991 and December 1997. In all cases, primary surgery achieved complete or optimal cytore-duction (residual lesion smaller than 2 cm). |
|  | Representativeness of the cases: 1 | Participants came from four Argentine gynecological services (Spanish, Naval, and German Hospitals of Buenos Aires City; Independence Clinic of Munro in the Province of Buenos Aires). |
|  | Selection of controls: 1 | Participants came from the same place. |
|  | Definition of controls: 1 | The 19 patients who refused the second-look laparotomy and IPCT were used as control group. Their average age was 58 years (range 49–65 years). |
|  | Comparability: 2 | The 51 patients underwent 6 cycles of endovenous chemotherapy (cisplatin 75–100 mg/m^2^ and cyclophospamide 600 mg/m^2^) with good tolerance. |
|  | Ascertainment of exposure: 1 | Samples for cytologic studies were obtained by peritoneal washings and multiple biopsies taken from suspicious areas or at random (15–25 biopsies). IPCT with HT was applied thereafter (cisplatin 100 mg/m^2^ in physiologic solution at 41–43°C). |
|  | Same method of ascertainment for cases and controls: 1 | Patients were followed up by means of trimestrial clinical evaluation and serum CA125 dosage, semestrial chest X ray and abdominopelvic ultrasonography, and an annual computer tomography scan. Median follow-up was 73 months (range 24–134 months). |
|  | Non-response rate: 1 | The response rate of follow-up subjects exceeded 90%. |

| Author, year | score | Reasons |
| --- | --- | --- |
| Kim, Jin Hwi 2010 | Adequate case definition: 1 | Patients with histologically confirmed stage Ic–IIIc epithelial ovarian cancer and evidence of a clinical complete response after cytoreductive surgery and adjuvant chemotherapy were considered eligible. The inclusion criteria were as follows: age younger than 75 years; a histologically- or cytologically confirmed diagnosis; performance status 0–2 (GOG); adequate cardiac, renal, hepatic, and bone marrow function; no evidence of residual lesions at 2nd-look laparotomy; and informed written consent. |
|  | Representativeness of the cases: 1 | Data were collected retrospectively on 43 patients (HIPEC-paclitaxel group: n=19, control group: n= 24) undergoing 2nd-look laparotomy between December 1991 and January 2004 at Kangnam St. Mary’s Hospital in Seoul, Korea. |
|  | Selection of controls: 1 | The survival rates were compared with 24 patients treated with conventional therapy (control group). |
|  | Definition of controls: 0 | Control group treated with conventional therapy. |
|  | Comparability: 2 | There was no statistically significant difference in clinicopathologic variables between the two groups. |
|  | Ascertainment of exposure: 1 | The perfusion circuit was set up with approximately 6 L of lactated Ringer’s solution and 175 mg/m^2^ of paclitaxel. The intraperitoneal temperature was monitored with thermometers inserted in the subphrenic space and perihepatic, perisplenic, and pelvic cavities, and maintained at 43–44°C for 90 min. Upon completion of the perfusion, the abdomen was irrigated with saline, then re-explored removing the cannulas, followed by routine abdominal closure. |
|  | Same method of ascertainment for cases and controls: 1 | All patients were followed every 3 months for 2 years, and every 6 months for the next 3 years. The follow-up included a clinical examination, serum CA 125 levels, and CT or positron emission tomography-CT. |
|  | Non-response rate: 1 | The follow-up was continued until March 2009. |

| Author, year | score | Reasons |
| --- | --- | --- |
| Cascales-Campos, Pedro Antonio 2014 | Adequate case definition: 1 | This article analyzed the data of 87 consecutive patients diagnosed with stage IIIC/IV ovarian cancer operated between December 1998 and July 2011. In every patient it was possible to achieve a complete cytoreduction of their disease. |
|  | Representativeness of the cases: 1 | During the period of time considered, a total of 87 patients were part of this analysis. Of the 87 patients, 52 (60 %) were treated after January 2008 and given HIPEC after complete cytoreduction of their disease. |
|  | Selection of controls: 0 | Of 87 patients, 52 were treated with HIPEC (paclitaxel 60 mg/m^2^, 60 min, 42°C). |
|  | Definition of controls: 1 | All patients were treated with the same systemic chemotherapy regimen consisting of a total of 6–8 cycles of a combination of platinum and taxanes scheme. |
|  | Comparability: 2 | No statistically significant differences in preoperative variables were found between patients treated with HIPEC against those not treated. A summary of the main variables of the series is reflected in Table 1. |
|  | Ascertainment of exposure: 1 | In patients treated with HIPEC (open technique), the cytostatic agent used was paclitaxel at doses of 60 mg/m^2^ diluted in 3 L of 1.5 % dextrose, keeping an average intraperitoneal temperature of 42 °C and a constant flow of 0.5–0.7 L/min for a total of 60 min. |
|  | Same method of ascertainment for cases and controls: 1 | The main objective of the study was the analysis of disease-free interval... Regional relapse-free survival was estimated from the date of surgery, using Kaplan–Meier’s analysis method. |
|  | Non-response rate: 1 | The median disease-free survival in patients treated with HIPEC at the time of the closing of the database for analysis was not met. |

| Author, year | score | Reasons |
| --- | --- | --- |
| Mendivil, Alberto A. 2017 | Adequate case definition: 1 | From October 2012 until September 2015, 95 histologically confirmed advanced stage ovarian, fallopian tube or primary peritoneal cancer patients who underwent optimal (<1 cm) debulking surgery and six cycles of primary, intravenous weekly paclitaxel (80 mg/m²) and day 1 carboplatin (AUC 6) chemotherapy, every 28 days, were initially considered to receive HIPEC. |
|  | Representativeness of the cases: 1 | Ultimately, we identified 69 advanced stage ovarian cancer patients (Group A) who underwent cyto-reductive surgery with intravenous chemotherapy and HIPEC. Alternatively, 69 advanced stage ovarian cancer subjects (Group B) who comprised the historical control group, were treated with cyto-reductive surgery and intravenous chemotherapy. |
|  | Selection of controls: 1 | This article retrospectively selected advanced stage ovarian cancer patients who underwent cyto-reductive surgery and six cycles of intravenous paclitaxel (175 mg/m²) and carboplatin AUC (5 or 6) every 21 days from April 2008 until November 2014 with whom to compare the HIPEC patients. The control subjects originated from the same institution and were treated by the physicians from the current investigation. |
|  | Definition of controls: 1 | Alternatively, 69 advanced stage ovarian cancer subjects (Group B) who comprised the historical control group, were treated with cyto-reductive surgery and intravenous chemotherapy. |
|  | Comparability: 2 | The two patient groups’ demographic and clinical characteristics were similar (P > 0.05). |
|  | Ascertainment of exposure: 1 | The patient was administered heated intraperitoneal carboplatin chemotherapy at a dose of AUC 10; the chemotherapy was mixed in 2500 cc of normal saline and added to the inflow fluid at a temperature of 41.5 °C; temperature measurement was conducted via esophageal temperature probes (DeRoyal; Powell, TN); the chemotherapy was circulated in the abdominal cavity for 90 min. |
|  | Same method of ascertainment for cases and controls: 1 | Progression-free survival (PFS) was defined as the length of time from the date of initial induction chemotherapy until clinical, radiologic, or CA-125 progression. Once disease progression was documented, the patient discontinued maintenance therapy and was treated at the physician’s discretion. Overall survival (OS) was defined as the time from the date of study entry until death with all causes of death treated equally. |
|  | Non-response rate: 1 | The overall median follow-up was 36 months (range 7–49) for the Group A patients and 44 months (range 6–78) for the group B subjects. |

| Author, year | score | Reasons |
| --- | --- | --- |
| Frankinet, Lisa 2023 | Adequate case definition: 1 | This was a retrospective analysis of 173 patients with CCRS with (n = 118) or without (n = 55) HIPEC treated at 12 French centers. Only patients having a completeness of cytoreduction (CC) 0/1 resection and a minimum of 5 years of follow-up were included. |
|  | Representativeness of the cases: 1 | All consecutive patients undergoing cytoreductive surgery (CRS) for advanced EOC (International Federation of Gynaecology and Obstetrics [FIGO] stages IIIC and IVA) in first line from 2005 to 2015 at these 12 specialized centers were collected. Overall, 173 patients treated by CCRS alone (n = 55), CCRS and open HIPEC (n = 56), or CCRS and closed HIPEC (n = 59), or method not specified (n = 3) were included in this study. |
|  | Selection of controls: 1 | HIPEC was performed systematically for all patients except those treated at the four centers that did not perform HIPEC. |
|  | Definition of controls: 1 | CCRS alone (n = 55) |
|  | Comparability: 2 | More patients in the CCRS-alone group had FIGO stage 4 disease (p < 0.001), serous histology (p = 0.002), CC-1 resections (p = 0.020) and prior laparoscopic surgery (p = 0.001), while more patients in the CCRS+HIPEC group had prior incomplete CRS (p = 0.004) (Table 1). |
|  | Ascertainment of exposure: 1 | The following HIPEC regimens were used for non-mucinous tumors: Cisplatin 75 mg/m² for 90 mins; Cisplatin 50 mg/m² + mitomycin C 15 mg/m² for 90 mins; Cisplatin 50 mg/m² + adriamycin 15 mg/m² for 90 mins. The target intra-abdominal temperature was 41–43 °C. |
|  | Same method of ascertainment for cases and controls: 1 | Recurrence was diagnosed according to the Gynecologic Cancer InterGroup (GCIG) criteria. |
|  | Non-response rate: 1 | Among the remaining 257 patients who were treated by CCRS following six or more cycles of systemic chemotherapy, 84 patients with inadequate follow-up were excluded. |

| Author, year | score | Reasons |
| --- | --- | --- |
| Karanikas, Michail 2024 | Adequate case definition: 1 | The files of the patients with newly diagnosed ovarian cancer from 2000 until 2020 were retrospectively reviewed. The files of patients that had previously undergone therapeutic surgery or of those who had undergone incomplete cytoreduction were excluded from the study. |
|  | Representativeness of the cases: 1 | From 2000 until 2020, 151 female patients, mean age 61.95 ± 12.34 years, with peritoneal carcinomatosis of epithelial ovarian cancer that did not receive NACT underwent complete (CC-0) or near-complete (CC-1) cytoreduction. Seventy-nine patients were treated with CRS plus HIPEC, while 72 were treated with CRS alone. |
|  | Selection of controls: 0 | Treated with cytoreduction alone. |
|  | Definition of controls: 0 | CRS alone. |
|  | Comparability: 2 | Patients treated with CRS plus HIPEC were younger in age (p = 0.033), were in better performance status (p = 0.021), presented increased morbidity (p = 0.001), and underwent more frequently retroperitoneal lymph node resection (p < 0.001). |
|  | Ascertainment of exposure: 1 | Patients treated with CRS plus HIPEC were younger in age (p = 0.033), were in better performance status (p = 0.021), presented increased morbidity (p = 0.001), and underwent more frequently retroperitoneal lymph node resection (p < 0.001). |
|  | Same method of ascertainment for cases and controls: 1 | The follow-up included physical examination, thoracic and abdominal CT-scan or MRI or PET-CT scan, hematologic and biochemical examinations, and tumor markers (CEA, CA-125). Recurrences and the sites of recurrence were recorded in detail. |
|  | Non-response rate: 1 | Follow-up data were available for all patients. |

| Author, year | score | Reasons |
| --- | --- | --- |
| Lei, Ziying 2025 | Adequate case definition: 1 | Patients were enrolled if they had a histologically confirmed diagnosis of epithelial ovarian cancer, no prior anti-tumor treatment before the operation, and no evidence of extra-abdominal metastasis. Patients with recurrent disease or other concurrent malignancies were excluded. |
|  | Representativeness of the cases: 1 | The data of patients with stage III epithelial ovarian cancer (n=789) treated at five high-volume gynecological medical centers between 2010 and 2017 were extracted from the database. |
|  | Selection of controls: 1 | Eligible patients with stage III epithelial ovarian cancer were stratified according to the presence of residual disease after surgery. PCS plus HIPEC versus PCS alone. |
|  | Definition of controls: 0 | PCS-alone group, n = 159. |
|  | Comparability: 2 | We employed the inverse probability of treatment weighting (IPTW) model to control for differences in baseline characteristics. |
|  | Ascertainment of exposure: 1 | Closed HIPEC was recommended to be simultaneously performed at the end of surgery. Cisplatin was administered via HIPEC with a dose of 50 mg/m² at 43 ± 0.1°C. |
|  | Same method of ascertainment for cases and controls: 1 | The Kaplan–Meier method was used to generate the survival curves, and the difference in OS between the groups was estimated with the log-rank test. |
|  | Non-response rate: 1 | After excluding 147 patients with recurrent disease, 4 patients complicated with other tumor types, and 54 patients with incomplete data, a total of 584 patients who met the enrollment criteria observed from January 2010 to May 2017 were included in this study. |
