# Supplementary material for: Hyperthermic intraperitoneal chemotherapy enhances survival outcomes in primary ovarian cancer following cytoreductive surgery: a systematic review and meta-analysis
Source: Front Oncol. 2025 Dec 3;15:1708318. doi: 10.3389/fonc.2025.1708318 (PMC12709118; doi:10.3389/fonc.2025.1708318)
Supplement: Supplementary file 10 [file Table4.docx]

Details of Risk of bias table score.

| Author, year | score | Reasons |
| --- | --- | --- |
| Antonio, Cascales Campos Pedro, 2021 | Random sequence generation (selection bias): low risk | Using a simple blinded randomization system carried out by an independent committee. |
|  | Allocation concealment (selection bias): low risk | Using a simple blinded randomization system carried out by an independent committee. |
|  | Blinding of participants and personne (performance bias): high risk | Underwent CRS alone (control arm) or CRS followed by the administration of HIPEC (experimental arm). Operative time: min (range) 220 (140–345) vs 300 (220–490); p < 0.001. |
|  | Blinding of outcome assessment (detection bias): unclear risk | No information provided on whether assessors of recurrence (e.g., radiologists) were blinded to the patient's group assignment. |
|  | Incomplete outcome data (attrition bias): low risk | Quote from Fig. 1: "0 Loss to follow-up." All patients who received treatment were included in the analysis. |
|  | Selective reporting (reporting bias): low risk | The trial was registered (NCT-02328716). All pre-specified primary and secondary outcomes were fully reported in the results. |
|  | Other bias: high risk | Due to a lack of funding to increase the participation of other centers in the recruitment, and especially due to the refusal of the patients to participate after hearing the results obtained in the Van Driel et al. trial, it was decided to suspend the recruitment of new patients after consulting the ethical committee. |

| Author, year | score | Reasons |
| --- | --- | --- |
| Lim, Myong Cheol, 2022 | Random sequence generation (selection bias): low risk | An independent statistical center randomly assigned the participants to the HIPEC group or the control group. |
|  | Allocation concealment (selection bias): low risk | The involvement of an independent center for randomization strongly implies that allocation was concealed until interventions were assigned. |
|  | Blinding of participants and personne (performance bias): low risk | The intervention is a complex surgical procedure. It is explicitly stated that "Participants were blinded to the group allocation." |
|  | Blinding of outcome assessment (detection bias): low risk | The use of these objective criteria (RECIST 1.1, GCIG CA125) for the primary outcome significantly reduces the risk of bias, even if the assessors were formally blinded or not. |
|  | Incomplete outcome data (attrition bias): low risk | The CONSORT flow diagram shows "3 Loss to follow-up" in the control group and "0 Loss to follow-up" in the HIPEC group out of 184 randomized participants. This low and balanced attrition rate is unlikely to introduce bias. |
|  | Selective reporting (reporting bias): low risk | The trial was registered (NCT01091636). The published report includes results for all pre-specified primary and key secondary outcomes in the methods section. |
|  | Other bias: unclear risk | The current treatment outcomes still need to be carefully interpreted because of the potential imbalance between 2 groups in terms of stage and type of primary treatment. |

| Author, year | score | Reasons |
| --- | --- | --- |
| Aronson, S. L., 2023 | Random sequence generation (selection bias): low risk | Randomisation was done centrally by the method of minimisation with the use of a web-based randomisation system. |
|  | Allocation concealment (selection bias): low risk | Randomisation was done "centrally" using "a web-based randomisation system". |
|  | Blinding of participants and personne (performance bias): low risk | The trial was open label, with no masking of patients, physicians, or data analysts to group allocation. |
|  | Blinding of outcome assessment (detection bias): low risk | Confirmed by imaging (according to RECIST version 1.1) or elevated CA125 concentrations, according to the Gynecologic Cancer InterGroup criteria." This use of pre-defined, objective measures for the primary outcome significantly reduces the risk of detection bias. |
|  | Incomplete outcome data (attrition bias): low risk | Follow-up was available for all patients except for one patient in the surgery group, who withdrew consent early after randomisation and was therefore censored at this point. |
|  | Selective reporting (reporting bias): low risk | The trial was registered (NCT00426257). The published report includes results for all pre-specified primary and secondary outcomes. |
|  | Other bias: low risk | The analysis accounted for stratification factors, and subsequent therapies after progression were balanced between groups, making it unlikely the survival benefit is explained by differential post-trial care. |
